# Supplementary material for: Ambient Air Pollution Exposure Association with Anaemia Prevalence and Haemoglobin Levels in Chinese Older Adults
Source: Int J Environ Res Public Health. 2020 May 5;17(9):3209. doi: 10.3390/ijerph17093209 (PMC7246731; doi:10.3390/ijerph17093209)
Supplement: Supplementary file 1 [file ijerph-17-03209-s001.pdf]

Supplementary Materials

Table S1. Results of 10-fold cross-validation for PM_1, $PM_{2.5}$, PM_{10} and NO_2.

Pollutants	Daily Model		Annual Averages	
	CV R^2	RMSE	CV R^2	RMSE
PM_1	55%	20.5 μg/m^3	75%	8.8 μg/m^3
$PM_{2.5}$	83%	18.1 μg/m^3	86%	6.9 μg/m^3
PM_{10}	78%	31.5 μg/m^3	81%	14.4 μg/m^3
NO_2	64%	12.4 μg/m^3	72%	6.5 μg/m^3

RMSE: Root mean square error; CV: cross-validation. PM_{10}, particulate matter with an aerodynamic diameter less than or equal to 10 μm; $PM_{2.5}$, particulate matter with an aerodynamic diameter less than or equal to 2.5 μm; PM_1, particulate matter with an aerodynamic diameter less than or equal to 1 μm and NO_2 nitrogen dioxide.

Table S2. Annual average concentrations and pair wise correlations of air pollutants.

	Spearman Correlation Coefficients (p value)			
Pollutants	PM_{10}	$PM_{2.5}$	PM_1	NO_2
PM_{10} (μg/m^3)	1	0.9248 (P < 0.001)	0.2835 (P < 0.001)	0.6146 (P < 0.001)
$PM_{2.5}$ (μg/m^3)		1	0.5191 (P < 0.001)	0.8182 (P < 0.001)
PM_1 (μg/m^3)				0.7928 (P < 0.001)
NO_2 (μg/m^3)				1

PM_{10}, particulate matter with an aerodynamic diameter less than or equal to 10 μm; $PM_{2.5}$, particulate matter with an aerodynamic diameter less than or equal to 2.5 μm; PM_1, particulate matter with an aerodynamic diameter less than or equal to 1 μm and NO_2 nitrogen dioxide.

Table S3. Characteristics of participants with haemoglobin blood samples data and without blood sample (BS) data.

Characteristics	Participants without BS Consent (N = 2756)	Participants with BS Consent (N = 10,611)	Total (N = 12,950)	p-value
**Age (±SD)**	63.7 (9.3)	63.0 (9.3)	63.2 (9.4)	0.001
**Sex**				<0.001
Female (%)	1554 (21.9)	5539 (78.1)	7093	
Male (%)	1202 (19.2)	5072 (80.8)	6274	
**Smoking**				<0.001
Never smoker (%)	1702 (19.7)	6941 (80.3)	8643	
Ever smoker (%)	712 (16.5)	3610 (83.5)	4322	
**Alcohol**				0.864
No (%)	1671 (18.7)	7283 (81.3)	8954	
Yes (%)	739 (18.5)	3252 (81.5)	3991	
**BMI (±SD)**	23.9 (4.0)	24.0 (5.1)	24(5.0)	
**Physical Activity**				<0.001
Low level (%)	869 (20.8)	3313 (79.2)	4182	
Moderate level (%)	709 (19.5)	2936 (80.5)	3645	
High level (%)	834 (16.2)	4303 (83.8)	5137	
**Education**				<0.001
No school (%)	1038 (18.5)	4562 (81.5)	5600	
Primary (%)	4311 (16.6)	217 (83.4)	2602	
Middle (%)	651 (24.0)	2057(76.0)	2708	
Higher education (%)	615 (25.8)	1770 (74.2)	2385	
**Fruit and Vegetable consumption**				0.009
Insufficient intake (%)	1307 (21.6)	4733 (78.4)	6040	
Sufficient intake (%)	1449 (19.8)	5878 (80.2)	7327	

**Fuel used at home**				<0.001
Clean (%)	1798 (24.4)	5580 (75.6)	7378	
Unclean (%)	946 (16.0)	4969 (84.0)	5915	
**Total Household income**				<0.001
≤15,000 (%)	1234 (18.5)	5442 (81.5)	6676	
>15,000 (%)	1485 (23.2)	4907 (76.8)	6392	
**Location of residence**				<0.001
Urban (%)	1061 (15.6)	5739 (84.4)	6800	
Rural (%)	1695 (25.8)	4872(74.2)	6567	

Table S4. Associations between 3-year pollutant moving averages and haemoglobin, by participant characteristics.

**Characteristics**	**PM_{10} β ± SE**	**$PM_{2.5}$ β ± SE**	**PM_1 β ± SE**	**NO_2 β ± SE**
Sex				
Male	−0.71 ± 0.09	−0.80 ± 0.12	−0.61 ± 0.09	−1.61 ± 0.10
Female	−0.63 ± 0.08	−0.74 ± 0.11	−0.72 ± 0.08	−1.84 ± 0.10
Interaction p-value	0.29	0.46	0.28	0.90
Age				
Below Median (70 years)	−0.65 ± 0.09	−0.77 ± 0.12	−0.64 ± 0.08	−1.65 ± 0.10
Above Median (70 years)	−0.66 ± 0.09	−0.70 ± 0.11	−0.63 ± 0.09	−1.69 ± 0.10
Interaction p-value	0.32	0.80	0.61	0.360
History of COPD/Emphysema				
Yes	−0.66 ± 0.21	−0.75 ± 0.27	−0.66 ± 0.15	−1.64 ± 0.28
No	−0.64 ± 0.06	−0.72 ± 0.08	−0.62 ± 0.06	−1.68 ± 0.07
Interaction p-value	0.74	0.71	0.96	0.81
Smoker				
Current tobacco use	−0.78 ± 0.10	−0.95 ± 0.14	−0.80 ± 0.10	−1.87 ± 0.12
No current tobacco use	−0.61 ± 0.08	−0.67 ± 0.10	−0.58 ± 0.07	−1.65 ± 0.09
Interaction p-value	**0.003**	0.05	**0.006**	0.12
Alcohol				
Ever	−0.74 ± 0.12	−0.79 ± 0.16	−0.69 ± 0.11	−1.69 ± 0.12
Never	−0.60 ± 0.07	−0.71 ± 0.09	−0.61 ± 0.07	−1.66 ± 0.09
Interaction p-value	0.08	0.30	**<0.001**	**0.01**
Total household income				
≤15000	−0.92 ± 0.08	−1.21 ± 0.11	−0.85 ± 0.08	−1.96 ± 0.10
>15000	−0.43 ± 0.12	−0.36 ± 0.15	−0.12 ± 0.10	−1.11 ± 0.12
Interaction p-value	0.06	0.99	0.16	0.29
Region				
South	−0.04 ± 0.20	0.25 ± 0.22	0.20 ± 0.10	−1.69 ± 0.98
North	−0.29 ± 0.06	−0.52 ± 0.08	0.19 ± 0.07	−0.30 ± 0.10
Interaction p-value	<0.001	<0.001	<0.001	<0.001

PM_{10}, particulate matter with an aerodynamic diameter less than or equal to 10 μm; $PM_{2.5}$, particulate matter with an aerodynamic diameter less than or equal to 2.5 μm; PM_1, particulate matter with an aerodynamic diameter less than or equal to 1 μm and NO_2 nitrogen dioxide. COPD: chronic obstructive pulmonary disease.

Table S5. Sensitivity analysis of the association between anaemia prevalence and moving averages of pollutants.

**Model Number**	**NO_2**	**PM_1**	**$PM_{2.5}$**	**PM_{10}**
† Model 1 [a]	1.51 (1.42–1.61)	1.26 (1.17–1.36)	1.24 (1.17–1.32)	1.14 (1.10–1.19)
†† Model 2 [b]	1.40(1.33, 1.48)	1.12 (1.05, 1.19)	1.11 (1.05, 1.16)	1.04 (1.01, 1.07)
†† Model 3 [c]	1.42 (1.35, 1.50)	1.15 (1.09, 1.23)	1.12 (1.06, 1.18)	1.05 (1.02, 1.08)

[a] Excluding participants (n = 3692) who had respiratory or cardiovascular comorbidity. [b] using 1-year average IQR increase. [c] using 5 years average IQR increase. † Model includes pollutant, age, sex, tobacco use, physical activity, education, body mass index (BMI), alcohol, place of residence,

household income, type of indoor fuel use, daily fruit and vegetables consumption. †† Model includes pollutant, age, sex, tobacco use, physical activity, education, BMI, alcohol, place of residence, household income, diabetes history, hypertension history, chronic lung disease history, type of indoor fuel use, daily fruit and vegetables consumption. IQR PM_{10}: 1 year: 29.60 μg/m³ and 5 year: 28.02 μg/m³. IQR $PM_{2.5}$: 1 year: 26.79 μg/m³ and 5 year: 26.69 μg/m³. IQR PM_1: 1 year: 23.70 μg/m³ and 5 year: 22.42 μg/m³. IQR NO_2: 1 year: 21.36 μg/m³ and 5 year: 22.79 μg/m³.

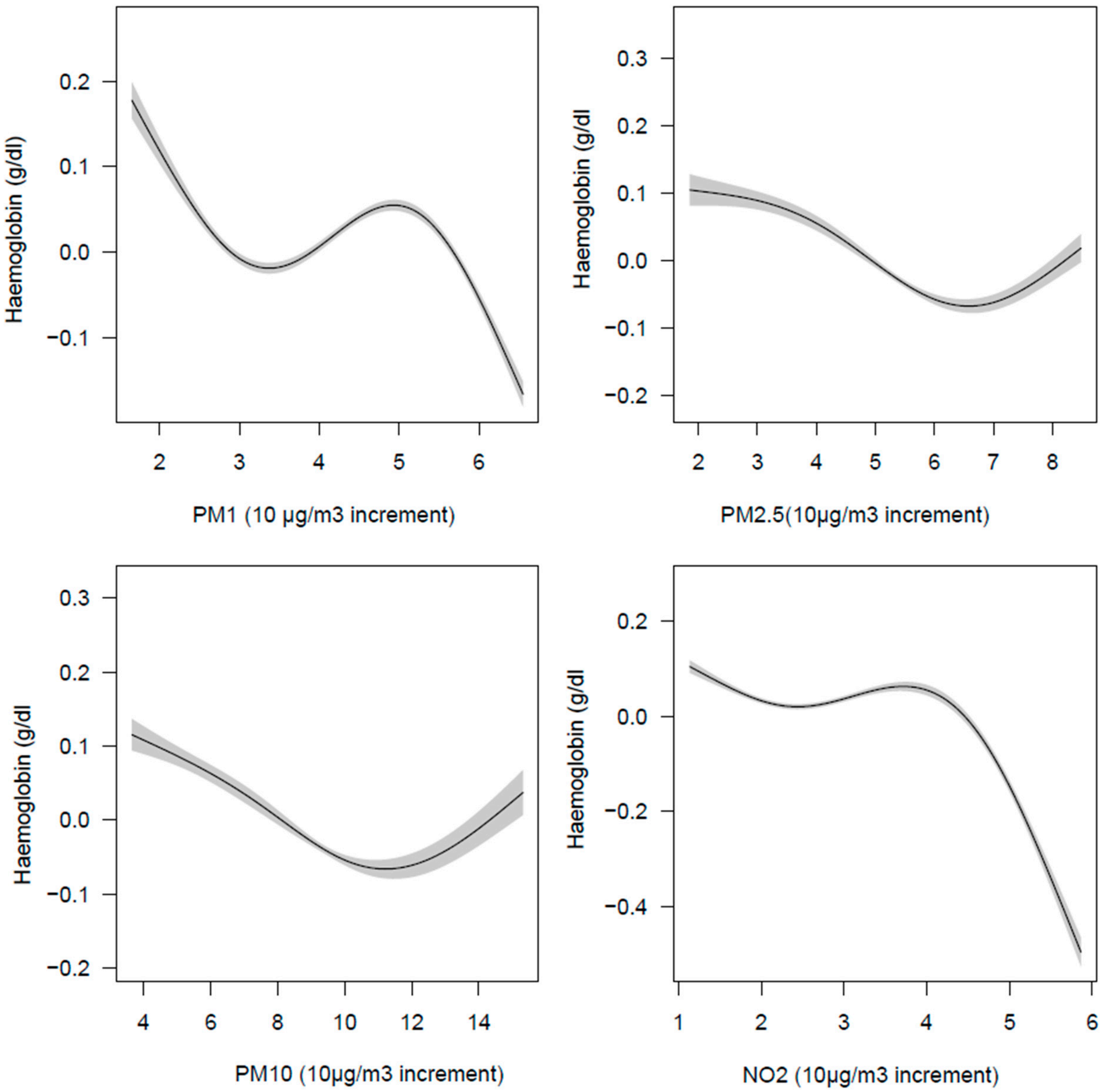

Figure S1. the association between a 10 ug/m³ increase of particulate matter (PM) and NO_2 and decrements in haemoglobin (g/dL). The shaded region indicates 95% CIs.
